# TeamWATCH: Visualizing development activities using a 3-D city metaphor to improve conflict detection and team awareness

**DOI:** 10.1371/journal.pone.0193562

**Published:** 2018-03-20

**Authors:** En Ye, Xin Ye, Chang Liu

**Affiliations:** 1 School of Electrical Engineering and Computer Science, Ohio University, Athens, Ohio, United States of America; 2 California State University San Marcos, San Marcos, California, United States of America; University of Bari, ITALY

## Abstract

The awareness of others’ activities has been widely recognized as essential in facilitating coordination in a team among Computer-Supported Cooperative Work communities. Several field studies of software developers in large software companies such as Microsoft have shown that coworker and artifact awareness are the most common information needs for software developers; however, they are also two of the seven most frequently unsatisfied information needs. To address this problem, we built a workspace awareness tool named TeamWATCH to visualize developer activities using a 3-D city metaphor. In this paper, we discuss the importance of awareness in software development, review existing workspace awareness tools, present the design and implementation of TeamWATCH, and evaluate how it could help detect and resolve conflicts earlier and better maintain group awareness via a controlled experiment. The experimental results showed that the subjects using TeamWATCH performed significantly better with respect to early conflict detection and resolution.

## 1. Introduction

As reported in [1], software engineers spend approximately 70% of their time on cooperative activities; thus, collaboration is essential for software development. At the same time, collaboration is also difficult since the intangible nature of software makes it challenging for software developers to create a common view among team members. A shared view of a software system can not only help developers better understand its complexity during collaboration but also enable them to know more about how their work relates to that of others within the context of the entire system. As the size and complexity of software systems produced in large-scale software development increase, it tends to incur a high degree of parallel development [2]. Therefore, coordination among different team members in a team or across teams working on different modules is necessary. With the progress of globalization and outsourcing, a growing number of software development projects are being geographically and temporally distributed, which increases the difficulty of collaboration since increasing the distance between team members usually leads to less effective communication [3,4].

The awareness of others’ activities has been widely recognized as essential in facilitating coordination in a team among Computer-Supported Cooperative Work (CSCW) communities. Awareness can be defined as “an understanding of the activities of others, which provides a context for one’s own activities” [5]. Much attention has been given to the importance of awareness in the coordination of software development due to the complexity and interdependency of software systems [6,7]. According to a two-month field study of “collocated” software developer teams at Microsoft [8], Ko et al. found that the most common and second most frequent information that these developers seek is coworker awareness, i.e., “what a developer’s coworkers have been doing”; another awareness information need, i.e., “how have resources I depend on changed”, was ranked as the third most common one. However, the above types of information are also regarded as two of the most frequently unsatisfied information needs. That is, collocated software developers have difficulty acquiring coworker and artifact awareness information. The importance of awareness regarding coworkers and artifacts and the inadequate tool support available to obtain it have been substantiated in two other similar studies on software developers at Microsoft [9,10]. Maintaining group awareness becomes even more difficult in distributed software development. A distributed team not only cannot take advantage of the ad hoc communication commonly used in collocated situations but also has to overcome the impact of working across different time zones, languages, and cultures [3,4]. As a result, team members may duplicate work or create conflict without knowing the status of others and the whole team, which may, in the end, impact the project schedule and cause delays with respect to project delivery [11].

Such problems can be seen in developers’ traditional usage of version control systems [12,13]. By using version control systems such as CVS [14] and SVN [15], developers can work independently most of the time. They usually become aware of others’ activities only when they perform operations (e.g., check-in, check-out, update) in the central repository. As a result, developers usually can only detect conflicts at these times. For example, when developer A wants to check-in a modified artifact, he finds that developer B has checked-in the same artifact with a different modification, which causes a conflict. At this time, the conflict may be too large; thus, resolving it (e.g., by merging changes, re-testing the artifact, etc.) can be a significant and time-consuming process. It may also be too late to resolve the conflict even if developer A can use some tools, such as CVS-Watch [14], to receive a notification as soon as developer B checks-in the modified artifact. To avoid these efforts in resolving conflicts, developers may rush to check-in their changes [16,17].

To solve these problems and thus enhance developers’ collaboration, tools need to be created to help developers acquire coworker and artifact awareness information easily, quickly and correctly. As suggested by Dourish and Bellotti, information of past activity and information of current activity were two facets of a single view of awareness information [7]. To provide awareness information regarding a coworker’s current status, such tools usually monitor developers’ workspace activities; thus, they are called workspace awareness tools. Gutwin et al. defined workspace awareness as “the collection of up-to-the-minute knowledge a person uses to capture another's interaction with the workspace” [18]. Gutwin et al. also referred to group awareness as “the understanding of who is working with you, what they are doing, and how your own actions interact with theirs” [19]. In this sense, group awareness is similar to workspace awareness, albeit with a closer focus on people instead of artifacts.

Workspace awareness tools aim to solve this problem of a lack of awareness among developers by providing developers with awareness information regarding which artifacts other developers are working on, what kinds of changes they are making, and whether their changes will affect local workspaces [20]. In the version control system example, conflicts could be detected and thus resolved earlier [21] if workspace awareness tools indicate to developer A that developer B is changing or has changed the same artifact he plans to change, no matter whether developer B’s change has been committed (i.e., in the central repository) or not (i.e., in the local workspace). Then, developer A can start a conversation with developer B to discuss their changes to avoid the potential conflict. Developer A can also choose to work on other artifacts first until developer B has committed his or her change.

To help software developers maintain group awareness and enhance their collaboration, we propose a workspace awareness tool based on a 3-D city metaphor called TeamWATCH (Team-based Workspace Awareness Toolkit and Collaboration Hub) [22,23]. TeamWATCH monitors developers’ activities in their local workspaces, version control repository, and bug tracking system. It then extracts and analyzes the corresponding awareness information and finally visualizes it in real time as a common view shared by the whole team using a 3-D city metaphor. With TeamWATCH, developers can obtain not only real-time awareness information (such as who is online, which tasks (i.e., bug items or feature requests in the bug tracking system) they are working on, and which artifacts they are manipulating) but also historical information (such as when the latest revision of an artifact was committed, who has changed an artifact most often, and how many revisions are contained in an artifact). It can support both workspace awareness that focus on artifacts and group awareness that focus on people.

## 2. Related work

To create such a workspace awareness tool, we first need to know what kinds of awareness information software developers are interested in and where and how they can gain this awareness information. According to [19], developers in open-source projects tend to maintain both a general awareness of the whole team and more detailed knowledge regarding team members that they plan to work with. First, developers acquire a broad awareness of the main team members working on their project and their areas of expertise. They obtain these kinds of information from three sources: mailing lists, text chat, and commit logs. Second, when developers plan to work in a particular area, they then try to gain more comprehensive knowledge regarding the people who have experience with that part of the codebase. Developers maintain this specific awareness by using a variety of information sources available during the project. These sources include the “maintain” field in the source tree, version control repository logs, issue trackers, help from senior developers, and the project document. They also ask related questions using the mailing list. To summarize, open-source developers maintain group awareness by manually “pulling” information from several information sources. This thesis also applies to commercial software developers based on the studies in [7–10,24].

Many tools have been developed to maintain group awareness. Some of them (e.g., COOP/Orm ([25], BSCW [26], Xia [27], and Augur [28]) provide awareness of activities based on information currently available in the repository; thus, they can only show changes that have already been committed and cannot offer real-time information regarding current activities in developers’ local workspaces. Other tools (i.e., workspace awareness tools) improve awareness by adding a visualization of up-to-date information regarding ongoing changes in developers’ local workspaces (e.g., Palantir [29–33], JAZZ [34], FASTDash [10], Workspace Activity Viewer [35], War Room [36], Scamp [37], CollabVS [38], Celine [39], TUKAN [40], State Treemap [41] and Crystal [42–44]).

From our analysis of the workspace awareness tools above, we have been able to make several observations:

- All the tools only extract awareness information from the version control repository and local workspaces. According to the observation in [19], i.e., that developers obtain awareness information from several sources, awareness information gained from only a single source is incomplete and thus may be incorrect or misleading. Meanwhile, if developers want to gain awareness information from other sources, they have to “pull” information by themselves, which incurs additional effort.
- All the tools, except the Workspace Activity Viewer, only present a developer’s real-time information regarding ongoing changes to artifacts (and may also provide the latest check-in info related to these artifacts). Although the Workspace Activity Viewer records the historical awareness information, it can only show developers the raw data, i.e., a snapshot of all ongoing changes to artifacts at a particular time. Actually, developers are also interested in historical information, especially the statistical results of this information. For example, in the beginning of this section, we mentioned that developers are interested in gaining more detailed knowledge about the people who have experience with a particular part of the code. If the statistical information regarding who has changed each artifact most often can be provided, developers can ask for help from this person when they have questions regarding this artifact since this person is more familiar with it.
- Among all the tools, some tools, such as Palantir and Jazz, display awareness information in a filtered view customized for individual developers, i.e., they only show information regarding activities related to artifacts that are either included in a developer’s local workspace or artifacts in which he has specifically registered interest. Other tools, such as the Workspace Activity Viewer, FASTDash and War Room, provide an overview of all the ongoing activities in a project’s code repository. FASTDash and War Room even create a layout representing the file structure of the project repository and use it to show information regarding changes made. Both visualizations are equally important. The filtered view can help developers solve the information overload problem, especially in a large-scale software project, while the overview layout can show the global state of the entire system so that developers can understand how their work relates with that of others within the context of the entire system.
- Among all the tools, Palantir, Workspace Activity Viewer, CollabVS, and Celine provide a filter mechanism to handle developers’ cognitive load, using which they can see only the changes that they are interested. In addition, probably due to privacy concerns, only CollabVS and Celine can enable developers to see others’ locally changing or changed code in their workspaces. This function may help developers solve conflicts quickly by enabling them to compare (or contrast) their own locally changed code with that of others when two or more developers are changing the same artifact.
- Among all the tools, Jazz, CollabVS and State Treemap provide communication functionality. FASTDash’s annotation function is actually asynchronous communication among team members. Jazz supports contextual communication, where developers can chat specifically regarding a certain artifact, and chat logs can be linked to the related code. Without this functionality integrated into the awareness tool, developers usually use other standalone communication tools to chat with others when they find conflicts or other problems regarding artifacts. This not only incurs the cost of context switch between awareness tools and communication tools but also makes the valuable chat logs easier to neglect or lose.
- Among all the tools, Palantir, CollabVS, TUKAN and Crystal also support the detection of indirect conflict through dependency analysis, while the others can only detect direct conflicts, i.e., the cases in which the same file is locally changed by more than one developer.

Comparisons of the workspace awareness tools mentioned above are shown in Table 1.

**Table 1:** Comparisons of workspace awareness tools.

Tool	Type	Awareness Info Source	Awareness Info Visualization	Awareness Info Filter	Integrated Communication Functionality	Conflict Detection	Evaluation
Palantir	Eclipse plug-in	Version control repository and local workspaces	2-D views customized for individual developers integrated into Eclipse	Allows developers to select the awareness info in which they are interested and only notifies developers regarding that info	N/A	Both direct and indirect conflict	Controlled experiment on mock software projects in a programming course
Jazz	Eclipse plug-in	Version control repository, local workspaces and presence	2-D views customized for individual developers integrated into Eclipse	N/A	Synchronous communications such as IM and Screen Sharing, asynchronous communications such as discussion board, and contextual communication based on the related source code	Direct conflict	Turned into a software product by IBM
FASTDash	Standalone tool	Version control repository, local workspaces and presence	2-D common view built on the file structure of the project repository shared by the whole team	N/A	Asynchronous communications such as annotations to the visualized file	Direct conflict	Field study on a software development team at Microsoft
Workspace Activity Viewer	Standalone tool	Version control repository and local workspaces	3-D common view shared by the whole team	Filters by developer and by artifact	N/A	Direct conflict	Visualization of simulated workspace activities for five open-source projects
War Room	Standalone tool	Version control repository and local workspaces	2-D common view built on the file structure of the project repository shared by the whole team	N/A	N/A	Direct conflict	Case study of a real software development company
Syde	Eclipse plug-in	Version control repository and local workspaces	2-D views customized for individual developers integrated into Eclipse	N/A	N/A	Direct conflict	Case study of two multi-developer projects in a programming course
CollabVS	An extension to Visual Studio	Version control repository, local workspaces and presence	2-D views customized for individual developers integrated into Visual Studio	Allow developers to select the awareness info they are interested in and only notifies developers regarding that info	Synchronous communications such as IM, audio/video, and screen sharing	Both direct and indirect conflict	User study on software engineers at Microsoft
Celine	Standalone tool	Version control repository and local workspaces	2-D views customized for individual developers	Applies different strategies to provide developers with only relevant info	N/A	Direct conflict	Daily used by engineers at STMicroelectronics
TUKAN	A plug-in for the Smalltalk system	Version control repository, local workspaces and presence	2-D views customized for individual developers integrated into Smalltalk	N/A	Synchronous communications such as IM and screen sharing, and asynchronous communications such as Email	Both direct and indirect conflict	Case studies of the authors’ research group and a software company for one week
State Treemap	Integrated into a platform named “MOTU”, which is an open- source project	Version control repository, local workspaces and presence	2-D views customized for individual developers	N/A	Synchronous communications such as IM and audio/video	Direct conflict	Used by a virtual team of architects
Crystal	Standalone tool	Version control repository and local workspaces	2-D views customized for individual developers	N/A	N/A	Both direct and indirect conflict	Case studies of nine open-source projects

## 3. Proposed solution

The goal of our work is to help software developers maintain group awareness to enhance their collaboration, which in the end improves their efficiency. To achieve our research goal, we propose creating a workspace awareness tool that can first extract and integrate awareness information from several sources and then visualize relevant information for developers in an appropriate 3-D form. We will briefly discuss the design and implementation of the tool (i.e., TeamWATCH) in the next two sections, with more details given previously in [22,23]. Then, we will focus on the evaluation of the tool, especially the tool’s efficiency in detecting and resolving conflicts earlier.

The tool will have the following features:

- It will extract awareness information from a variety of sources, which at least include the version control repository, local workspaces, and issue tracking system. These are the only two awareness sources that are based on actual manipulations of the project artifacts.
- It will provide developers both real-time awareness information and (statistical) historical awareness information and the way to highlight the information in which the developers are more interested.
- It will visualize awareness information in 3-D in two ways: a filtered view for individual developers and an overview layout for the whole team.

To build such a tool, we mainly consider three aspects:

## Information collection

What kinds of awareness information do we plan to extract from the version control system and issue tracking system? We plan to extract the following awareness information:

1. Presence awareness information
  - The status of a developer (busy, away, etc.)
  - The task that a developer is currently working on
2. Real-time awareness information
  - The artifacts each developer is changing or has changed in his or her local workspace compared with the latest version in the version control repository. The “changing” operation can represent any of the following actions: add, delete, rename, move, modify, update (i.e., check-out), and commit (i.e., check-in). This is more towards workspace awareness view focusing on artifacts.
  - The developers that are changing or have changed each artifact in their local workspaces compared with the latest version in the version control repository. This is more towards group awareness view focusing on developers.
3. History awareness information
  - Who has checked in each artifact most recently (or last)
  - Who has checked in each artifact most often
  - Who contributed most to the project, and who was most recently active
  - Which artifact has gone through the largest number of revisions
  - During which time period, which developers worked on the project most actively, and what were the changes made during this period

The workspace awareness information will also definitely include the details of each artifact changed by each developer, i.e., the version number if the artifact is checked-in, the time of the change, the person in charge of the change, and the size of the change (e.g., the number of changed lines).

### Information extraction

How do we extract and integrate awareness information from the version control system and issue tracking system? To extract awareness information, daemons will be created to monitor operations in the version control repository and issue tracking system and to store the extracted awareness information in the database. Since these two sources are mainly related to each other through artifacts, we can combine information from them together based on the artifacts. Integrating data regarding commits in the version control system and change requests in the issue tracking system can provide developers a historical view regarding which artifacts are related to the bug, who is responsible for it, which version of the artifact resolves it, and when.

### Information presentation

How do we present relevant awareness information to developers in an appropriate 3-D form?

Shneiderman suggested the following: “A useful starting point for designing advanced graphical user interfaces is the Visual Information-Seeking Mantra: overview first, zoom and filter, then details on demand” [45]. We try to apply this principle to the design of TeamWATCH visualization. First, a common overview of awareness information is visualized based on the file structure of the project repository. Second, animations are created to highlight active artifacts (i.e., all the files that have local changes, which have not yet been committed to the central repository, from the developers’ workspace). Third, filters are implemented to help developers quickly locate the artifacts in which they are interested. Meanwhile, developers can leverage the zoom function provided by the underlying 3-D platform. Finally, the detailed awareness information can be shown when developers hover their cursors over the artifact visualization. We will discuss the detailed visualization design in the following paragraphs.

**Fig 1:**
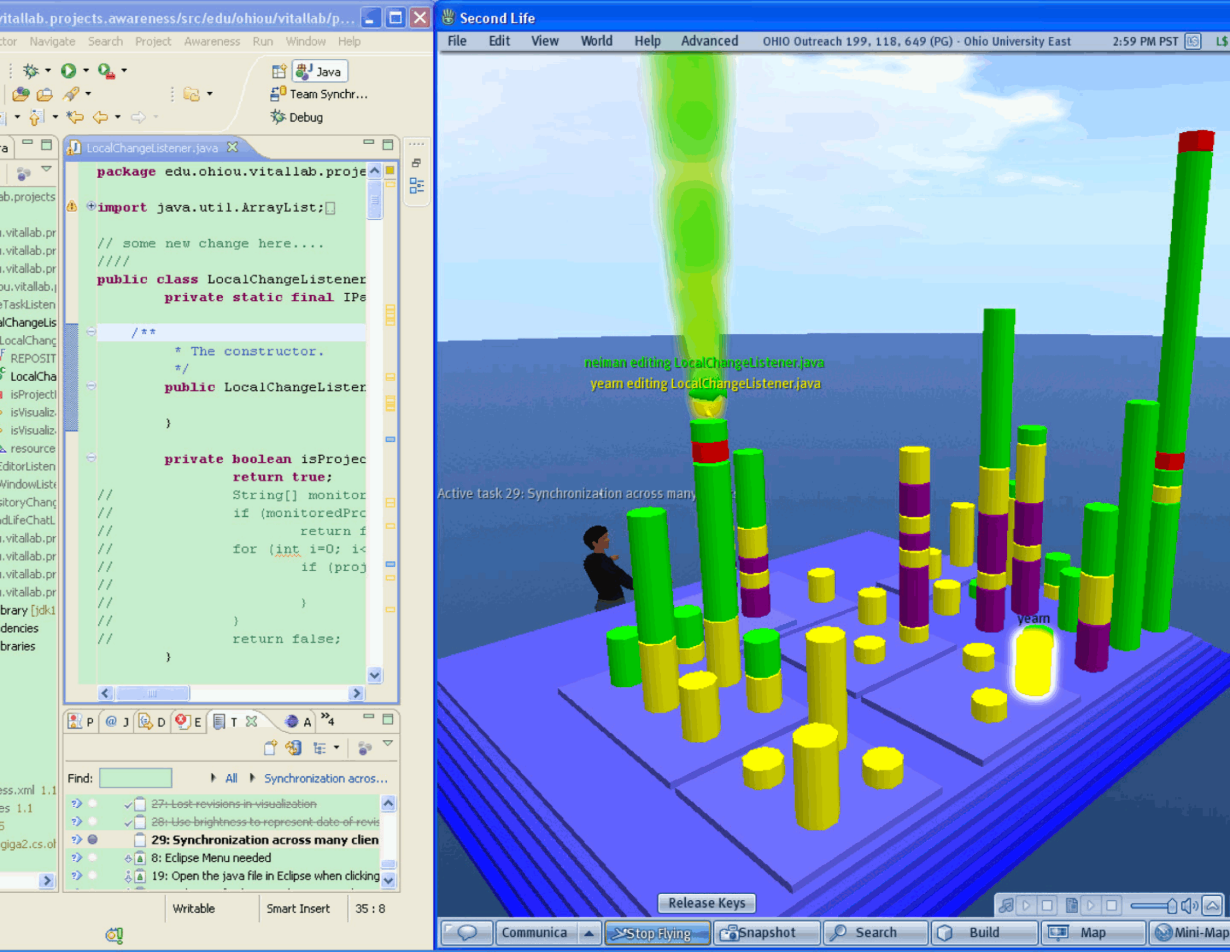
a) Eclipse; b) Second Life; c) Smoke representing active artifact, with the color representing the corresponding developer making local changes; d) Texts summarizing local changes made by the developers; e) Active task; f) Highlighted artifact.

TeamWATCH visualizes developers’ status through a buddy list and shows the task ID and summary (Fig 1D in Fig 1, which is a screenshot of the Second Life client of TeamWATCH) above the head of each developer’s avatar. This is a way to visualize presence information. It uses a 3-D city metaphor similar to the metaphor used in the CodeCity project [46] to visualize both real-time and historical artifact awareness information. As the visualization in CodeCity, TeamWATCH uses city buildings to represent files and city districts to represent folders, with the layout of the city representing the overall file structure of the software project. In TeamWATCH, buildings, shown as differently colored stacked cylinders, stand on top of city districts, shown as flat blue rectangular blocks (with color saturation representing the nested packages). Different from CodeCity, which visualizes class metrics (such as the number of methods in a class), TeamWATCH visualizes artifact awareness information. In addition, animations are used to highlight active artifact information since developers are more interested in it compared with historical information [10]. Animations are created when a developer makes local changes to an artifact. They will disappear after all its local changes have been committed or rolled back. By monitoring the animation, developers can learn about what other developers are doing, detect whether others are working on the same artifact, and take actions if necessary to avoid potential future conflict resolutions.

TeamWATCH visualizes a file as a stack of differently colored cylinders, just as a building has differently decorated floors. The height of the stacked cylinders indicates the number of revisions of the corresponding file. A single cylinder represents a revision, with its color denoting the author of that revision. If the same developer contributes successive revisions, the corresponding cylinders will be combined into one bigger cylinder, with its height equaling to the sum of the heights of all revisions. The stacked cylinders are sorted by date such that the most recent revision is always on top. In this way, developers can determine the number of revisions to a file based on the heights of the cylinders, the author of the latest version based on the color of the top cylinder, and who has committed the most revisions in total based on which color dominates the cylinders.

TeamWATCH uses animations to highlight active (or real-time) artifact information. When a file is changed locally, smoke, the color of which represents the developer making the change, will be emitted from the corresponding building (Fig 1C) and rise into the sky. Through this animation, developers can determine the popularity of a file and then decide whether to make changes to it, as they may encounter merge conflicts later due to the changes made by other developers to the same file. If more than one developer changes the same file, an entire building will shake, warning the team about a potential conflict.

The visualization mapping between the 3-D building and the software artifact is shown in Table 2.

**Table 2:** Mapping between a 3-D building and the project.

Attributes of the building	Attributes of the software artifact
Coordinates on a horizontal surface (X, Y)	Layout of a project (artifacts, including files and folders)
Height (Z)	Number of revisions to a file, ordered based on the revision time, with the latest one always on top
Floor	Revision
Shape (cylinder vs rectangular block)	Files vs folders
Color	The author of the revision
Transparency	Status (deleted or not)
Smoke emitted from the top of the building	Active artifact that is being changed locally by the developer corresponding to the smoke color
A building with lights on (glowing)	Highlighted artifact

With the current visualization, it may be difficult for developers to locate an artifact in which they are interested if the code base is large. Filters are created to solve this problem. TeamWATCH provides a revision number filter, revision time filter, author filter, and artifact filter. When the developers type any keyword in a filter dynamically, only the artifacts matching the search criteria are shown in the visualization, while other artifacts become invisible. For example, if the developers enter the keyword “notepad” in the artifact filter, then only artifacts whose name contains “notepad” are shown in the visualization. Developers can quickly revert to the normal state by removing the keywords in the filter. The filters are only applied to a local developer’s view, i.e., the views of other developers are unaffected by the filter operation. Therefore, the filters can be used to create a customized personal view for each developer without affecting the views of other developers. To obtain detailed information of an artifact, developers can hover their cursors over the corresponding visualization. They can also click a building to make it glow to highlight an artifact (Fig 1F).

## 4. Implementation

### 4.1 TeamWATCH

**Fig 2:**
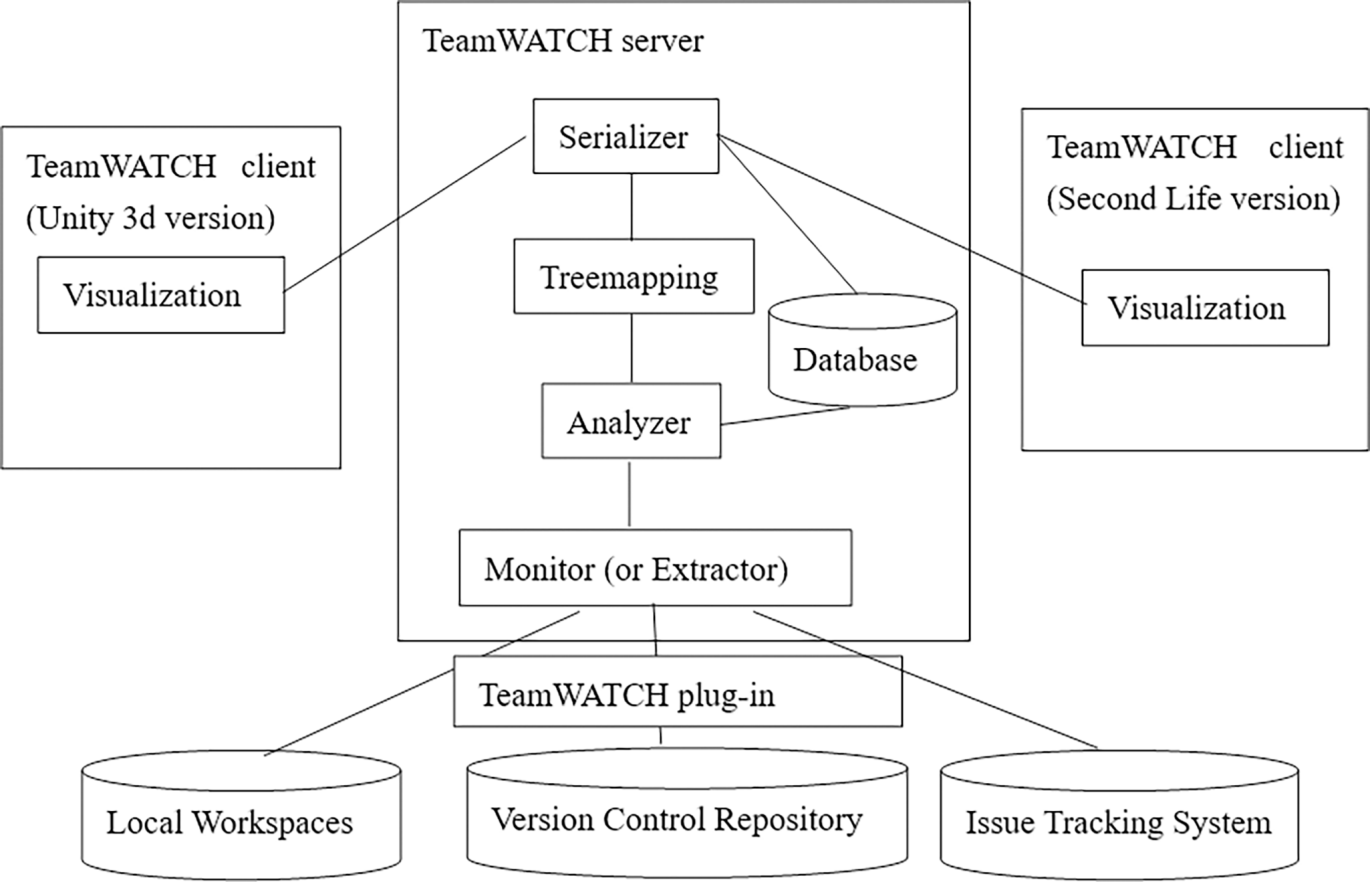
The TeamWATCH architecture diagram.

TeamWATCH is implemented in a client-server architecture, as shown in Fig 2. TeamWATCH mainly consists of three components: TeamWATCH server, TeamWATCH visualization client, and TeamWATCH plug-in. The server side is implemented as a Java Web service. The Monitor (or Extractor) on the TeamWATCH server receives the project’s real-time awareness information from the developers’ local workspaces and the project’s historical awareness information from the version control repository and issue tracking system via the TeamWATCH plug-in (currently only available in Eclipse). The TeamWATCH plug-in is built on top of the Eclipse CVS plug-in, to obtain notifications regarding developers’ operations in the local workspace and central repositories, and the Eclipse Mylyn plug-in, to determine developers’ current tasks in the issue tracking system. Then, it sends the awareness information to the extractor. The extractor also supports directly extracting a project’s historical log information from a repository by sending commands to the repository through SCM (Software Configuration Management) clients such as Subversion. Either way, the awareness information extracted from the client contains details such as the author, revision time, and files that are being or have been changed for every revision of the project. The Analyzer accepts the raw awareness data extracted by the Extractor as the input, then formats it and calculates the statistical results, such as how many check-ins each developer has contributed to the project, how many revisions each file has gone through, etc., before sending the information to the Tree-mapping component. The Tree-mapping component maps the project’s structure information onto 3-D coordinates using the Quantum Treemap algorithm [47], then creates visualization data for each artifact based on the mapping strategy described in Chapter 3. The tree-mapped output is then serialized into a string by the Serializer component, which is the final output of the TeamWATCH server. The TeamWATCH client invokes the TeamWATCH Web service to obtain the serialized string for visualization. To avoid repeated analysis, both the original awareness information and the final generated serialized string information are stored. When any new changes are made to the project, only new awareness information needs to be calculated and mapped onto the existing 3-D layout. For example, if the serialized 3-D layout result of a project with 735 revisions is stored in the database, when someone commits a new revision 736, only the information of revision 736 needs to be processed and added to the existing information of revisions 1 to 735 stored in the database.

The TeamWATCH client was implemented as a standalone application using either Second Life (SL) [48] or Unity3D. The client side of TeamWATCH is mainly the visualization of the project’s real-time and historical changes. It obtains information via a web service request to the server and presents the final visualization to the users as a common view.

The first TeamWATCH client was implemented based on a modified open-sourced SL client viewer [22] (aka SecondWATCH). SecondWATCH takes advantage of SL’s avatar-based virtual world to simulate the developer’s workspace, leveraging its 3-D object building feature to create 3-D objects representing software artifacts; it then utilizes SL’s various communication functionalities (text chat, Instant Message (IM), group message, and voice chat) to provide interaction between team members. Later, we found that the SL client is heavy-weighted, having a dependency on the SL server, which was not stable and has performance and latency issues, especially when visualizing large-scale software projects. This would affect the usability of the TeamWATCH tool. Therefore, we implemented another client using Unity3D, and the evaluation of the TeamWATCH tool discussed in this paper is based on the Unity3D client. The Unity3D client can run on multiple platforms, including Windows PCs, Mac computers, and iOS devices.

### 4.2 Application

**Fig 3:**
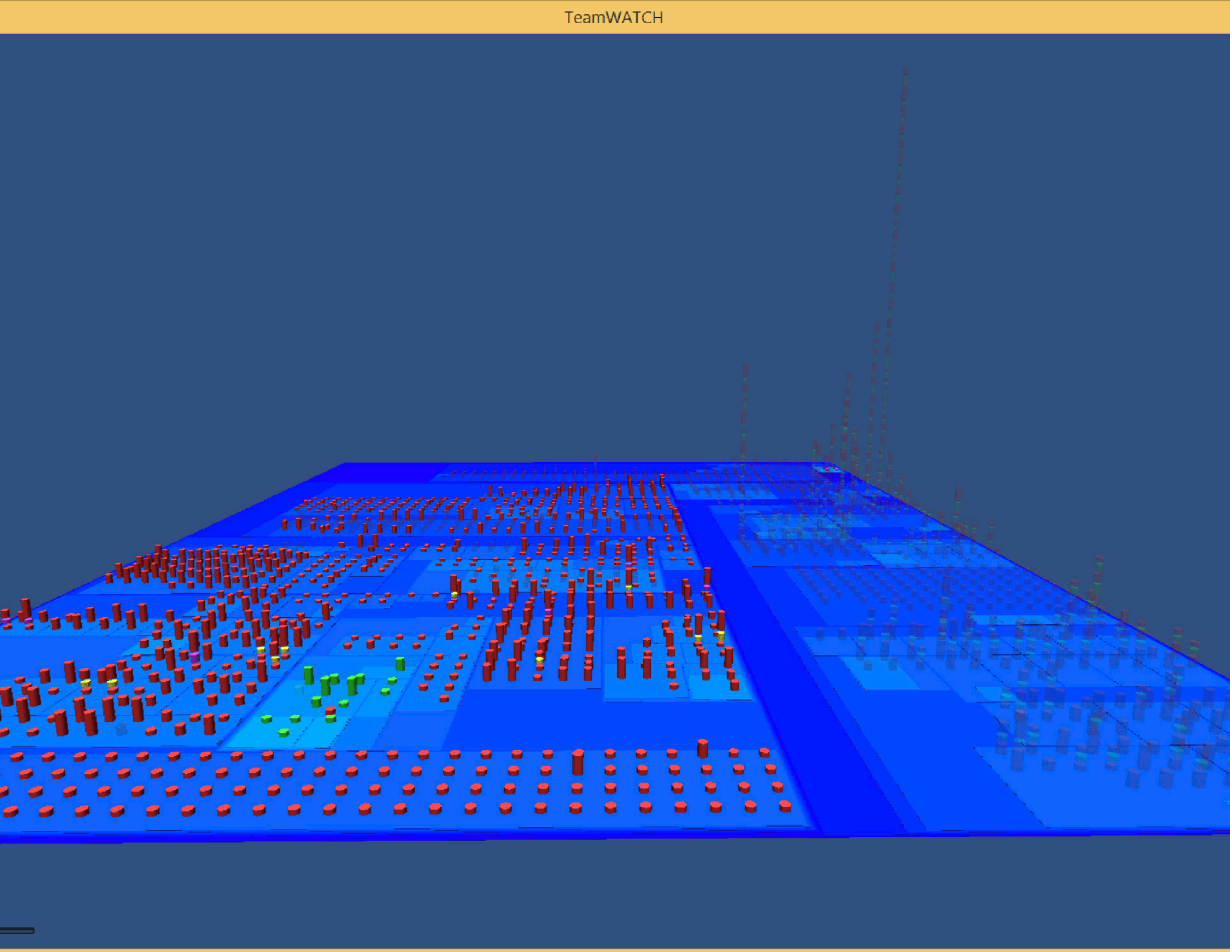
Visualization of the Notepad++ project generated by the TeamWATCH Unity3D client.

An introduction video for TeamWATCH is available on YouTube (http://youtu.be/xPDilTwfySU). The TeamWATCH visualization can be presented on a second personal display for individual developers or on a large display in a shared workspace for the whole team, which is very common in the software industry. TeamWATCH was used as our workspace awareness tool to visualize its own development process (see Fig 1) while we were developing it. It has also been successfully applied to visualize the historical awareness information of real-world open-source projects including Notepad++, jEdit, Firebird, Hugin, OpenNMS, FreeMind, and GUJ. Descriptions and screenshots of these visualizations, as well as those of the TeamWATCH software and user guide, are available on the VITAL Lab website (http://vital.cs.ohio.edu/?page_id=1340). A screenshot of the awareness information visualization of the Notepad++ project using the TeamWATCH Unity3D client is shown in Fig 3.

### 4.3 Comparisons with existing workspace awareness tools

Compared with existing workspace awareness tools such as Palantir [29], JAZZ [34], and FASTDash [10], TeamWATCH is different in the following ways. First, TeamWATCH not only extracts awareness information from the version control repository and local workspaces but also from the bug tracking system. Second, TeamWATCH visualizes both real-time awareness information and historical awareness information together using a 3-D city metaphor, which provides a common view that enables developers to refer to the information they need within the context of the whole team’s work. Third, TeamWATCH highlights active artifacts that are being changed locally and potential conflicts via eye-catching animations and combines the common view of the whole team with the customized personal view of an individual developer through the use of local filters. Thus, it can enable developers to quickly locate the artifacts in which they are interested and obtain the corresponding awareness information they want.

## 5. Evaluation

To answer the questions of whether and how TeamWATCH helps to maintain group awareness, improve development efficiency, and thus enhance team collaboration, controlled experiments have been conducted. The experiments conducted to evaluate the ability of TeamWATCH to help increase the correctness of and reduce the time needed to search for software historical information from a version control repository were discussed in our previous work [23]. In this paper, we discuss an experiment designed to evaluate the efficiency of TeamWATCH in detecting and resolving potential conflicts based on the real-time awareness information, and compare two groups of subjects: those who use TeamWATCH and those who do not use it. In the experiment, subjects from both groups were randomly divided into teams of two, each of them working together to finish a few tasks from a text editing project hosted in a CVS repository. Subjects’ opinions about the tool and their objective performance data were captured and evaluated. Since the study involved human subjects, it was approved by the Institutional Review Board (IRB) at Ohio University, with the approval number 14X104, before the experiments were conducted. The participants provided their written informed consent to participate in this study, which was also approved by the IRB.

### 5.1 Experimental design

#### 5.1.1 Research questions and hypotheses

The experiment was intended to address the following research question:

**Q1: Do the developers who use TeamWATCH detect and resolve potential conflicts earlier**, **thus encountering fewer merge conflicts, compared with the developers who do not use TeamWATCH?**

The null hypotheses and alternative hypotheses corresponding to the research question is listed in the Table 3.

**Table 3:** Null hypotheses and alternative hypotheses.

Null hypotheses	Alternative hypotheses
H1o: Using TeamWATCH does not help developers detect and resolve potential conflicts earlier	H1a: Using TeamWATCH helps developers detect and resolve potential conflicts earlier

The research question is answered through quantitative analyses below.

#### 5.1.2 Independent and dependent variables

In the controlled experiment, the tool used by the subjects to finish the tasks is the only independent variable because the intention is to test the null hypotheses and to answer the research questions by comparing the effectiveness and performance of TeamWATCH with respect to a baseline in software project development. Therefore, TeamWATCH and a baseline are the two choices for the independent variables.

To evaluate the general assumption that workspace awareness obtained with the support of a tool can improve developer efficiency, this study compares the performances of subjects using workspace awareness tools such as TeamWATCH with the performances of subjects who do not use any form of awareness tool to perform the same tasks. Therefore, the baseline chosen for the experiment should be none of any form of awareness tool. To represent users developing software under their normal working environments but without a workspace awareness tool, Eclipse IDE was chosen as the baseline of the experiment.

The dependent variables of the experiment are as follows: (1) the number of potential conflicts detected and resolved to avoid merge conflicts, and (2) subjects’ feedback regarding whether and how the tools helped them to maintain group awareness.

General feedback from the subjects regarding their feelings during the experiment was also recorded after all the experimental tasks were completed. Such feedback serves as important complementary material for the qualitative analysis of whether and why subjects were satisfied or unsatisfied using TeamWATCH.

#### 5.1.3 Contextual project

In the selection of the project, we started with an open-source Java project and came up with a few coding tasks based on it. However, during the trial of the experiment, we found that the subjects mostly only focused on finishing their own coding tasks without taking the time to check their team member’s status. This might be because the recruited subjects were undergraduate or graduate students who did not have much Java programming skills. Although the coding tasks are straightforward, it still took them some time to figure them out. Additionally, the subjects’ programming skills varied, which may have affected the validity of the experimental results. Therefore, we decided to switch to a text editing project, in which the subjects functioned as editors working on a few text editing tasks. Compared with the coding tasks, the students’ text editing skills were sufficient for the experiment and did not vary enough to create a bias.

Another reason we used a text editing project in the experiment was to evaluate early conflict detection and resolution, as editing text and editing code are very similar activities that trigger similar conflicts. In our previous work [23], when evaluating the historical awareness information visualization, we chose a real software project and designed experimental tasks based on the awareness information in which developers are most interested.

The text editing project is based on a book titled "EFF's Guide to the Internet" (formerly “The Big Dummy's Guide to the Internet”), which was written by the staff of the Electronic Frontier Foundation in 1994. It was chosen as the project for this experiment because of the following reasons: (1) The topic of the book, i.e., the Internet, is well known to the subjects, who are computer science students, and thus should not create any bias toward subjects who are more familiar with the content. (2) It went through a couple of editions, thus providing opportunities for the experiment designer to not only create a number of revisions in the repository based on the actual number of book revisions but also come up with related text editor tasks.

#### 5.1.4 Subjects

In the controlled experiment, computer science undergraduate and graduate students with ages ranging from 19 to 35 were recruited to serve as the subjects. Twenty-four subjects were recruited from a class dual-listed for both undergraduate and graduate CS students. Originally, we had planned to divide the 24 participants equally into two groups. However, two of the machines prepared for the treatment group had the issue of running TeamWATCH before the experiment began; thus, we had to move two students to the control group. Among the participants, ten were randomly selected and assigned to the treatment group, which used TeamWATCH. Their average time of experience with version control systems such as CVS, SVN, and GIT was approximately six months (based on the survey results from 8 subjects). Fourteen were placed in the control group. The control subjects also had an average of 6 months of experience using version control systems (based on the survey results from 12 subjects), similar to the treatment group. In the treatment group, 6 out of 8 participants who finished the pre-experiment survey had less than one year of experience with 3-D games. Background info of the control subjects regarding 3-D gaming was not collected because it was irrelevant; the control subjects did not use any 3-D tools.

#### 5.1.5 Tasks

The design of the experimental tasks aimed at evaluating the effectiveness of TeamWATCH compared to that of traditional IDEs in enabling developers to remain aware of software awareness information and to detect potential conflicts. The experimental tasks were designed to simulate the awareness information required by developers and potential conflicts they may encounter during their team activities.

To test our hypotheses, answer the research questions, and evaluate the TeamWATCH tool, five tasks were designed for the subjects to work on. Each task consisted of one subtask that involved answering questions regarding the historical information of the project and one or two subtasks that required making changes to the files in the repositories. Four of these five tasks required two subjects in a team to work on the same file, thus creating the possibility of direct conflicts. The details of each task are attached in the S1 Table.

#### 5.1.6 Procedures

The experiment was conducted simultaneously for both groups; however, since one room could not fit all the subjects from both groups, we reserved two rooms and assigned the control group to one room while the experimental one to the other. Before the experiment, subjects were given a CVS and Eclipse assignment, and a tutorial of TeamWATCH to get familiar with them. The subjects were randomly assigned to either the treatment group or the control group. Then, the subjects from both groups were randomly assigned to a team of two. Every subject in both groups was aware of being part of a team of two, and who is their team member. To make the experiment simulate a real distributed software development environment and to make it fair for both groups, the subjects were required to use the IM tool (i.e. Google Chat) to communicate with each other about the project status and to collaborate to finish the tasks instead of just talking and coordinating verbally. All these instructions are explained in the same experiment sheet given to both groups. All participants were asked to answer some survey questions related to their familiarity with the source control system, 3-D games, team development, etc. They also needed to set up the experiment by checking-out the text editing project from the CVS repository in Eclipse and by installing and running a video capture software to record their experimental process. The treatment group also needed to set up TeamWATCH to visualize the repository of the text editing project. During this period, participants were allowed to ask questions about problems they encountered.

During the experiment, participants were required to work on the same set of five tasks (with the two subjects in each team being assigned five different tasks). The treatment group was asked to use Eclipse plus TeamWATCH (i.e., the Unity3D client in this experiment) to finish the task, while the control group was asked to use Eclipse to do the same. Subjects in both groups were provided with answer sheets, on which they were asked to write down their answers for each subtask regarding the historical information of the project, and they were also asked to check-in the changes for the text editing subtasks. Subjects were asked to check-in at least once per task.

After finishing the five tasks, all participants were then asked to fill out a survey about the overall experiment experience. Then, subjects from the treatment group were asked to fill out another survey to provide their feelings regarding the use of TeamWATCH in this experiment with respect to whether they felt TeamWATCH is more helpful in maintaining group awareness, etc. Subjects from the control group were not asked to answer these questions because they were not exposed to TeamWATCH. Subjects from both groups were encouraged to provide additional feedback regarding their experiences in this experiment, which is included in the discussion presented later in this section.

### 5.2 Experimental results

Overall, we collected the following experimental data

1. The video recording of each subject’s experimental process via the video recording software running on the experimental computer. For the experimental group, we successfully recorded nine (out of ten) subjects’ experimental processes, approximately 366 minutes in total; for the control group, we collected thirteen (out of fourteen) subjects’ video recordings, approximately 311 minutes in total, although the recordings of some subjects in the control group were very short (less than ten minutes), indicating that the whole process was not captured. One subject from each group ran into an issue related to video recording; thus, we could not capture their experimental processes.
2. Each subject’s checked-in changes for the five tasks in the CVS repository
3. The chat logs between subjects in the same team during the experiment
4. Each subject’s answers to the survey questions before and after the experiment

In the following three subsections, we first present a detailed example of how the subjects in the experimental group performed during the experiment. Then the experimental results regarding the efficiency in tasks related to potential merge conflicts and feedback regarding the maintenance of group awareness in general are compared and discussed.

#### 5.2.1 A detailed example of the experimental process

We take developer 6 of team 3 as an example to show how he performed while completing the first two tasks. Figs 4–11 below are screenshots taken from the video recording of developer 6’s experimental process.

**Fig 4:**
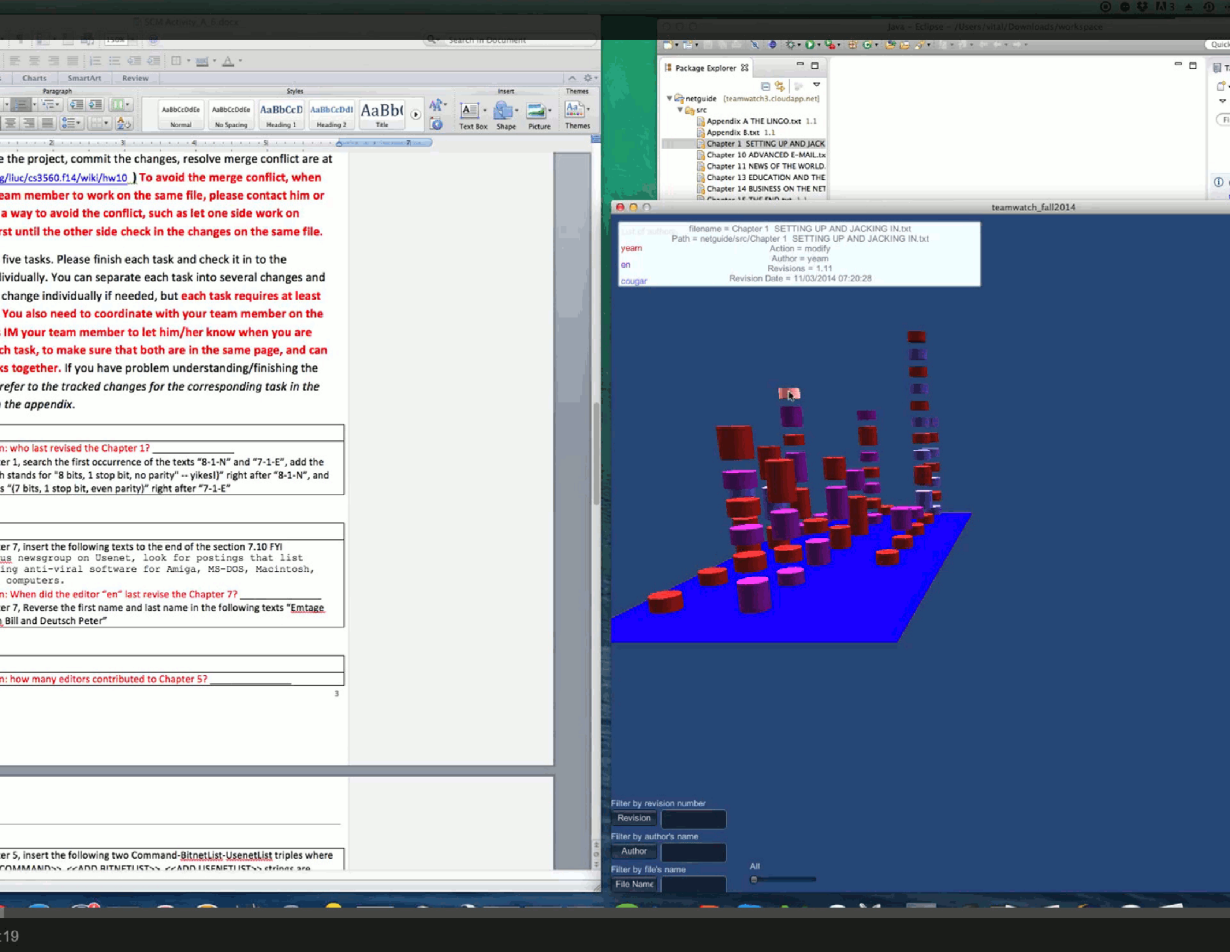
Developer 6 uses TeamWATCH (shown on the right side) to determine the answer to the CVS question (shown on the left side) in task 1.

**Fig 5:**
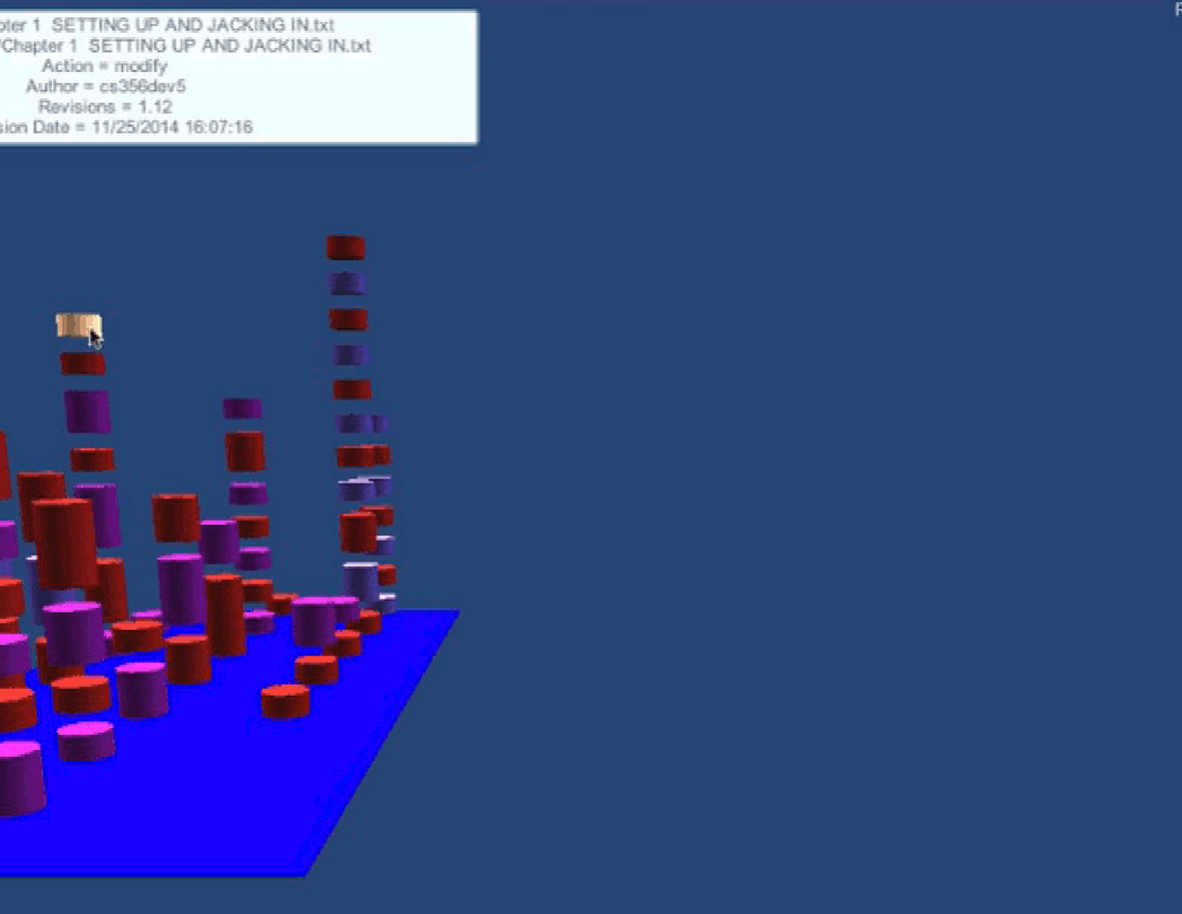
Developer 6 notices the committed changes from developer 5 via TeamWATCH.

**Fig 6:**
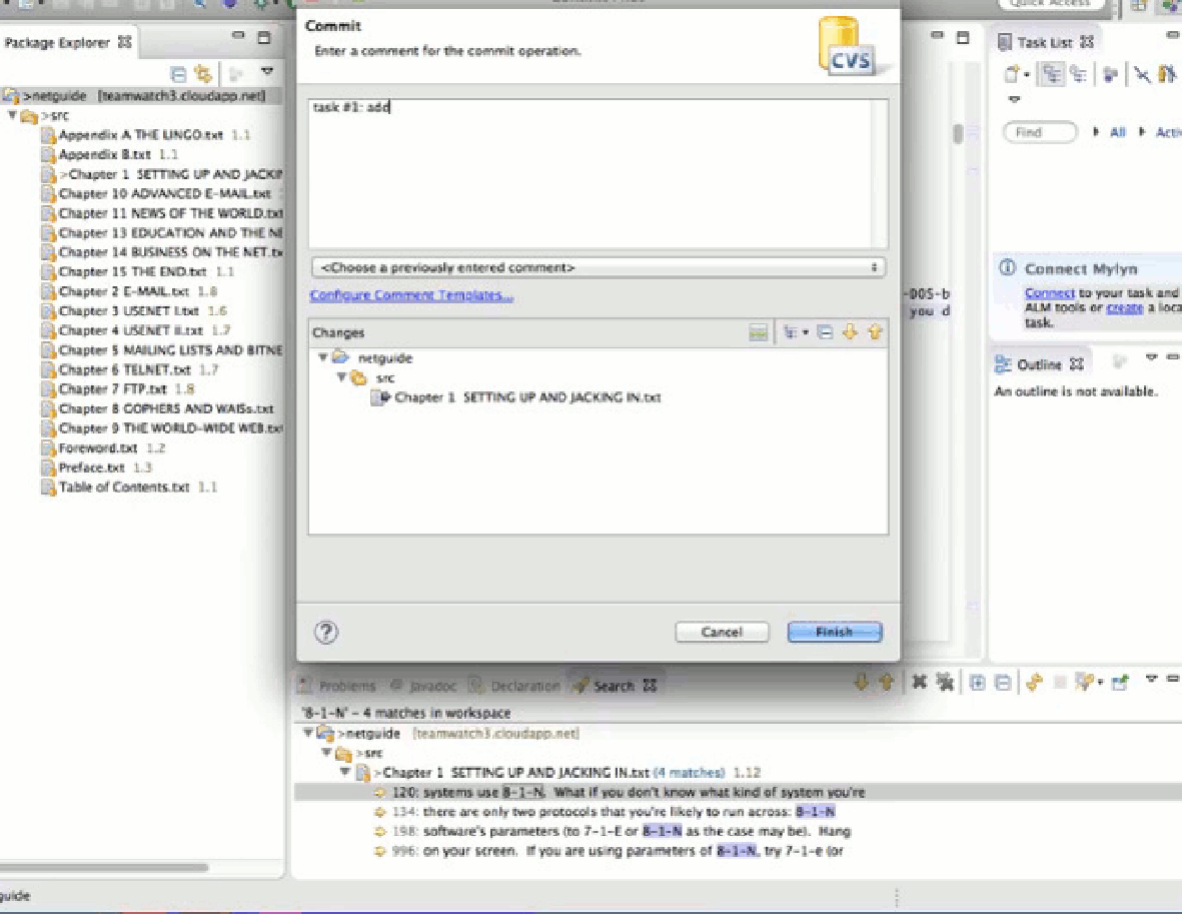
Developer 6 checks-in his local changes for task 1.

**Fig 7:**
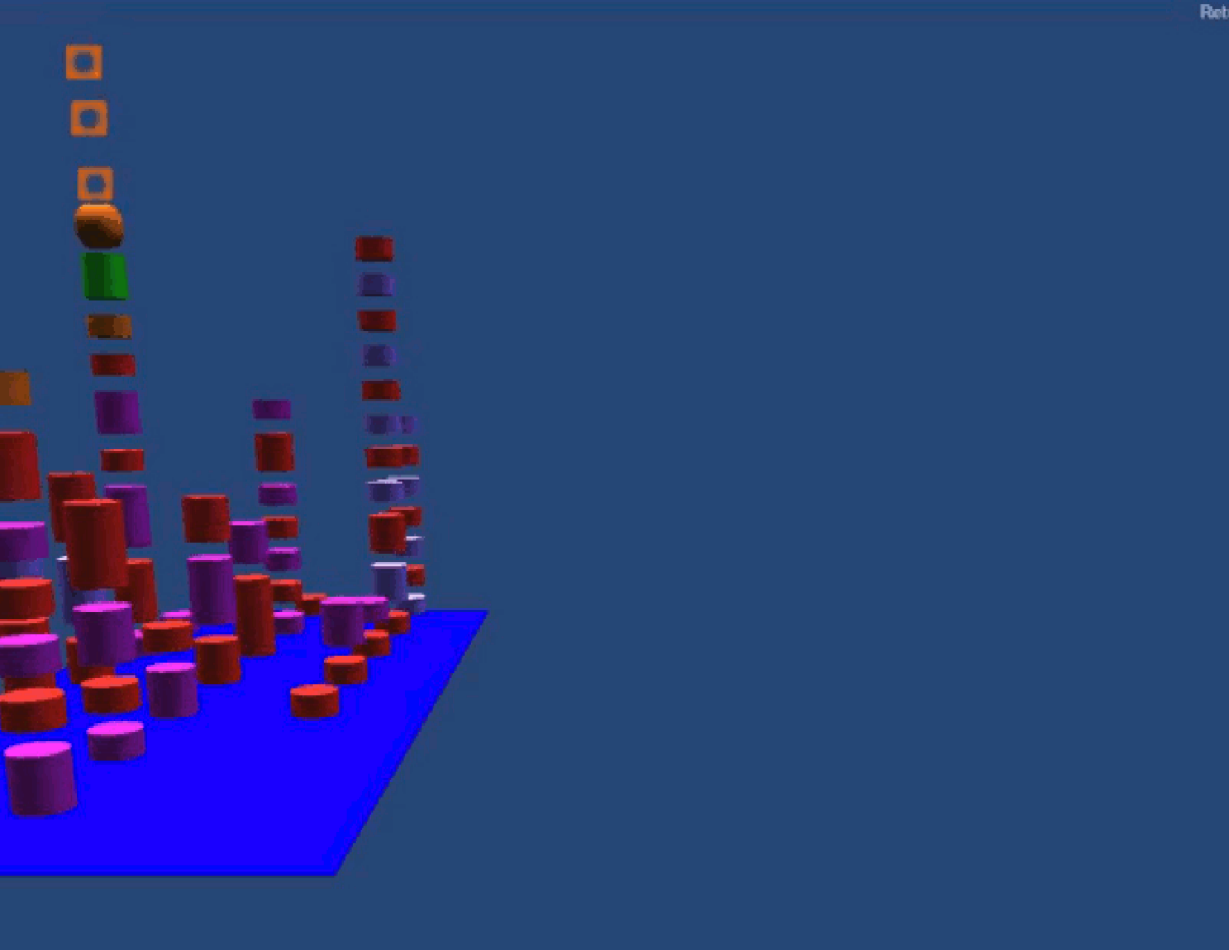
Developer 6 notices that developer 5 is also working on task 2.

**Fig 8:**
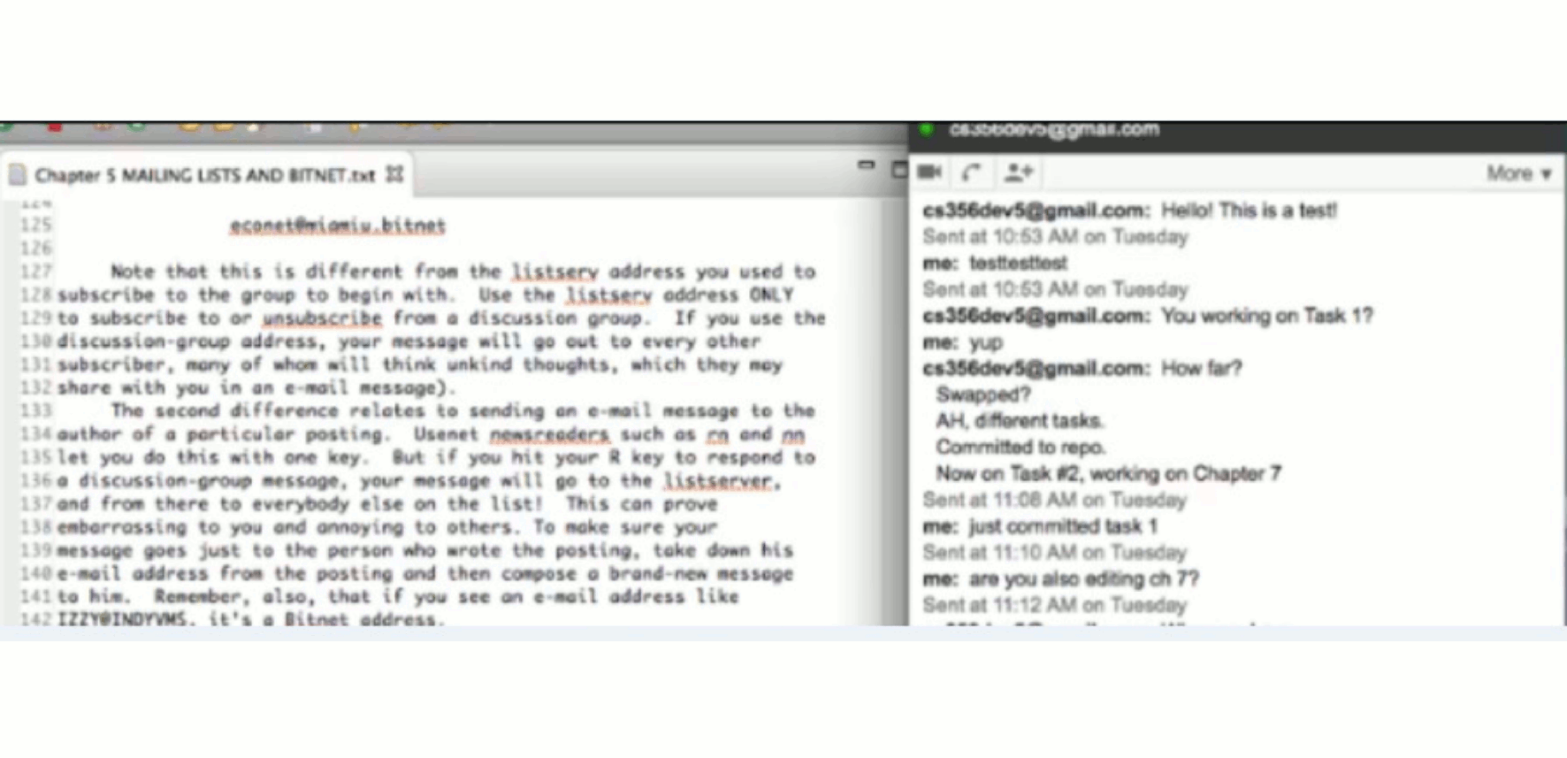
Developer 6 sends an IM message to developer 5.

**Fig 9:**
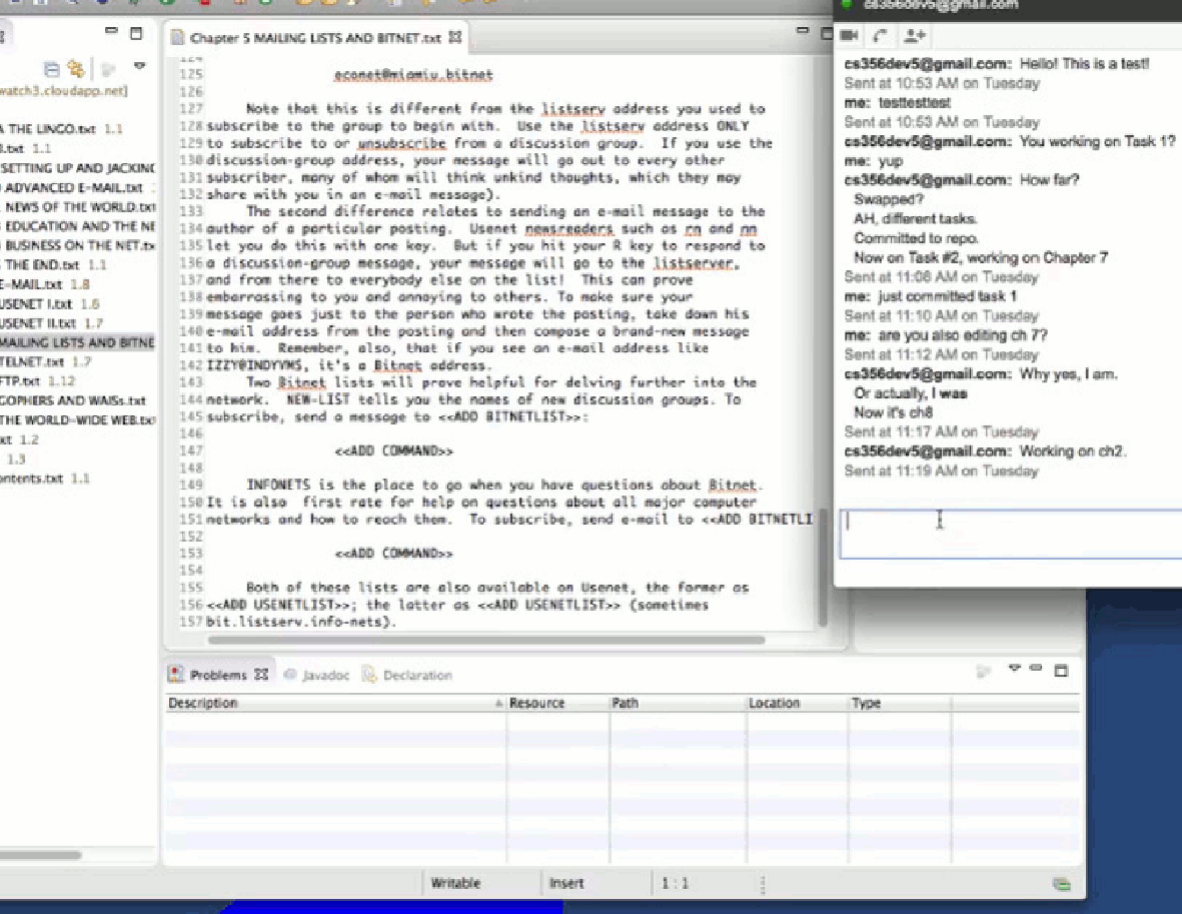
Developer 6 receives confirmation from developer 5 regarding the status of task 2.

**Fig 10:**
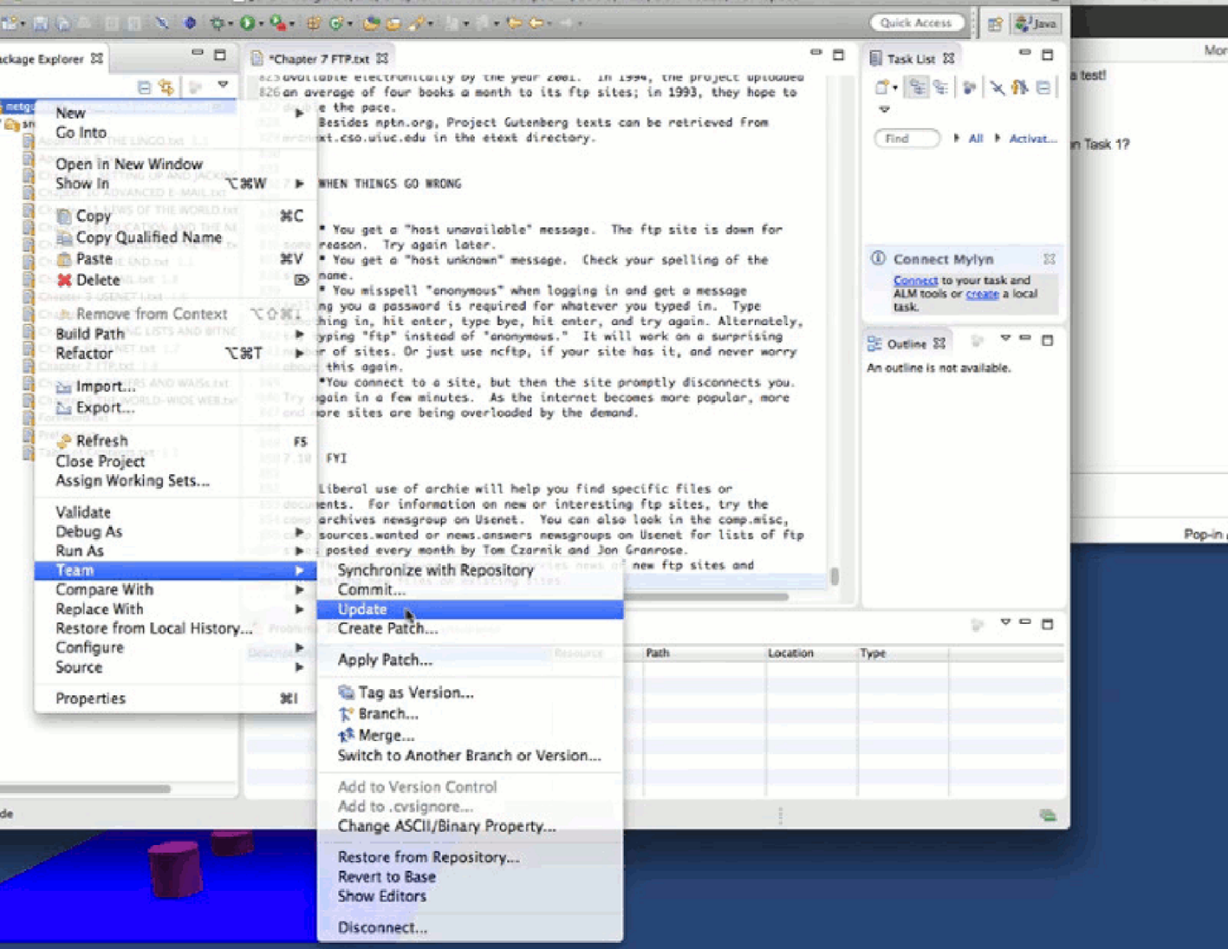
Developer 6 syncs with the repository to get the latest changes.

**Fig 11:**
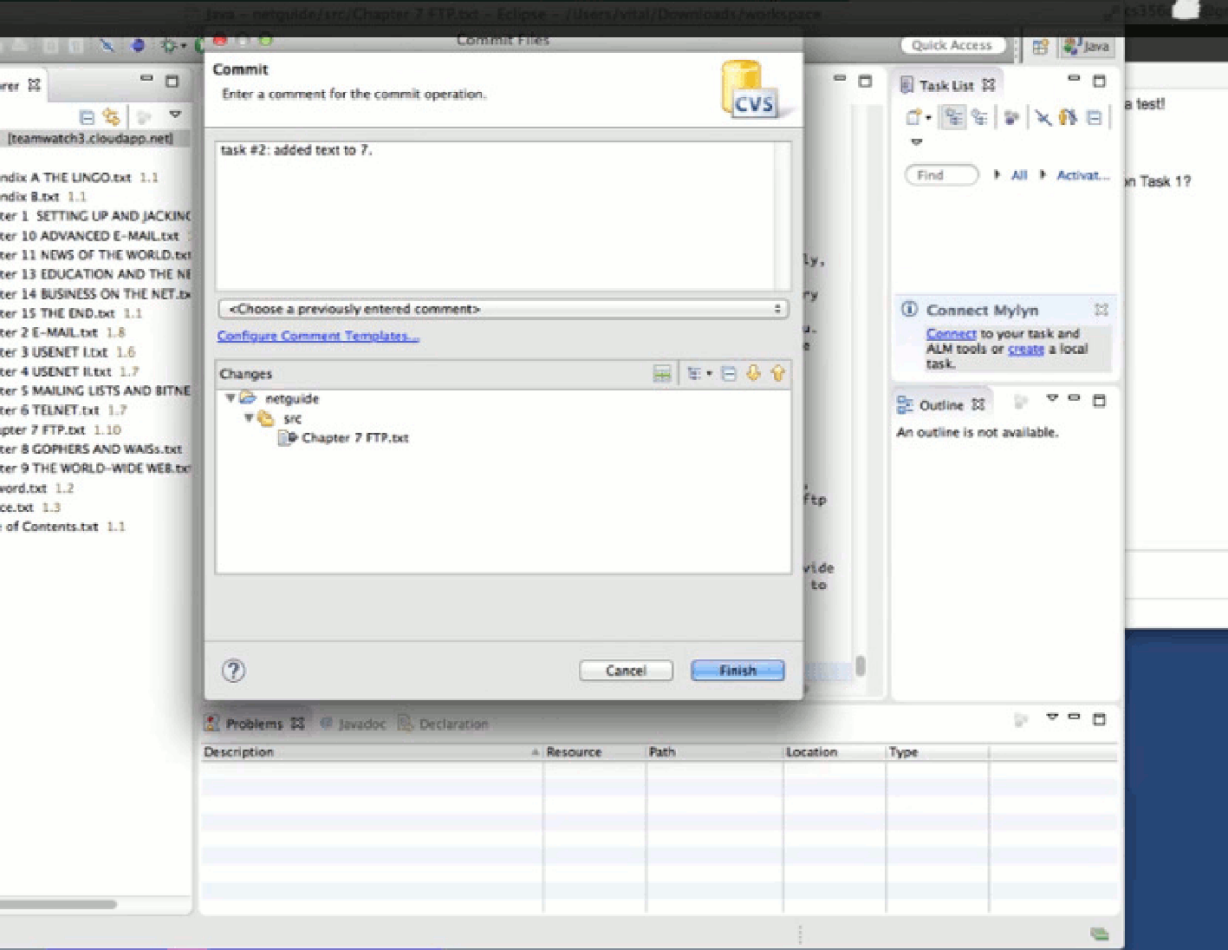
Developer 6 finally commits his changes to Chapter 7, i.e., task 2.

Developer 6 started the experiment by answering the first CVS question in the first task, i.e., who last revised Chapter 1; he obtained the answer by checking the top cylinder of the visualization of Chapter 1 revisions in TeamWATCH, as shown in Fig 4.

Developer 6 then began working on the text editing subtask in task 1, i.e. *in the Chapter 1*, *search the first occurrence of the texts “8-1-N” and “7-1-E”*, *add the texts “(which stands for "8 bits*, *1 stop bit*, *no parity"—yikes*!*)” right after “8-1-N”*, *and add the texts “(7 bits*, *1 stop bit*, *even parity)” right after “7-1-E”*, He then might notice the appearance of a new cylinder on top of the visualization of Chapter 1, i.e., newly committed changes from developer 5 for task 1.

Developer 6 synced with the repository to get the latest changes to Chapter 1 from developer 5. He then finished task 1 and checked-in his changes.

Developer 6 began working on task 2 (i.e., Chapter 7) and might notice that developer 5 was also working on task 2 based on the visualization.

Developer 6 sent an IM message to developer 5 to discuss the status of task 2.

Developer 6 received confirmation from developer 5 that he had checked-in his changes to Chapter 7, i.e., task 2.

Then, developer 6 synced with the repository to get the latest changes to Chapter 7 from developer 5.

Developer 6 finally committed his changes to Chapter 7, i.e., task 2.

#### 5.2.2 Analysis of conflict early detection and resolution

The primary objective of this experiment was to evaluate whether TeamWATCH can help developers detect and resolve potential conflicts early enough to avoid merge conflicts. Therefore, in the experimental results, we currently only checked whether there were merge conflicts and did not differentiate whether the merge conflicts were resolved before checking-in the changes. Among the experimental data that we collected, this analysis was mainly based on the video recordings of the experiment and the check-ins in the repository.

**Fig 12:**
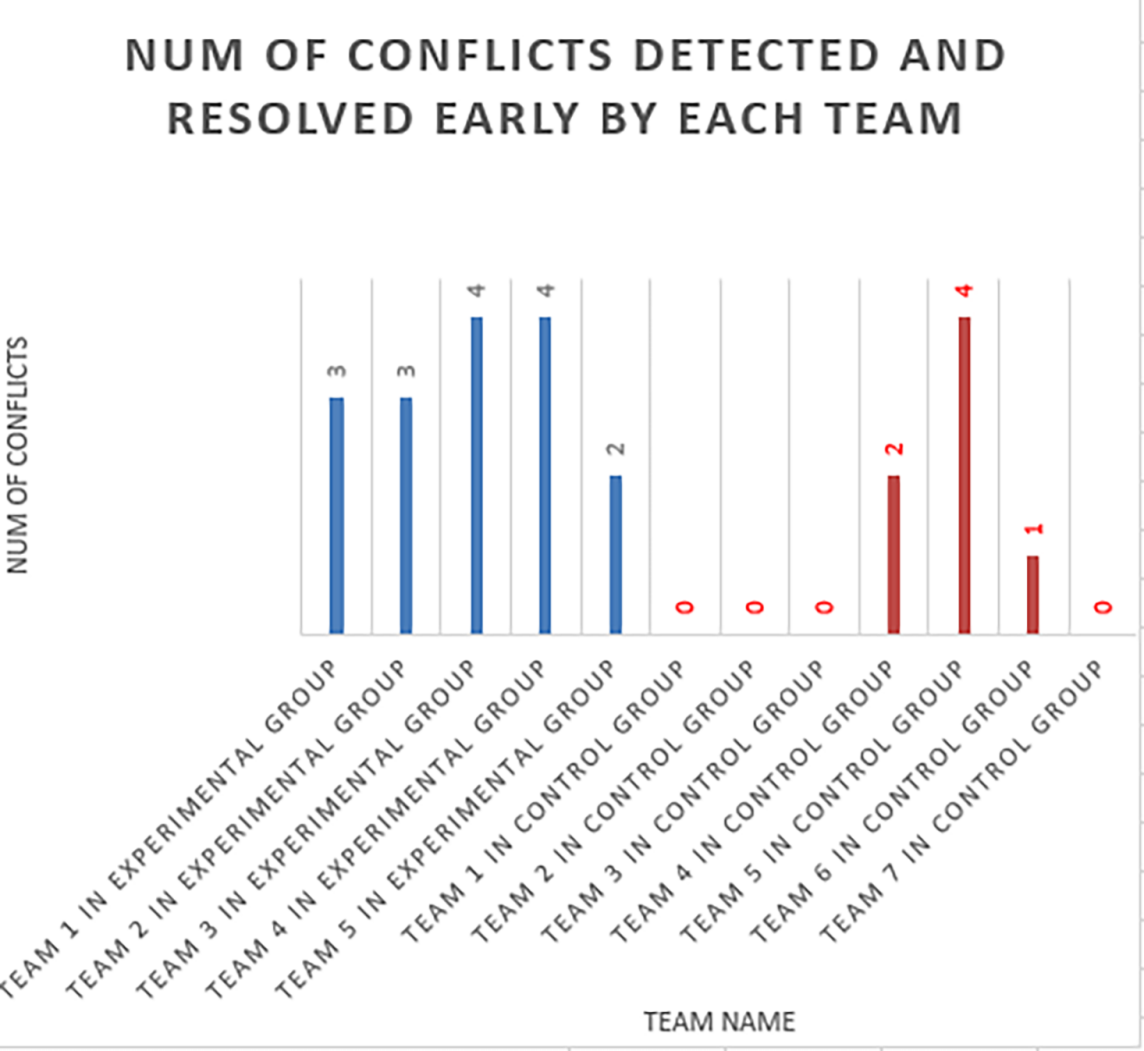
The number of conflicts detected and resolved early by each group.

There were four conflicts in total in the ten tasks (five per team member) for each team. The number of conflicts detected and resolved early (i.e., those that did not turn into merge conflicts) by both the experimental group and the control group is shown in Fig 12. A non-parametric statistical test, The Mann-Whitney U test, was applied in this comparison to assess significance levels of both groups since the probability distributions of results from both groups were unknown, as is common in this type of study. U is the Mann-Whitney U test statistic, which is then used to determine P, which in turn indicates whether a result is statistically significant. A result is significant at p< = 0.05 using the Mann-Whitney U test (the U-value is 4.5; the critical value of U at p≤0.05 is 5). Therefore, we could reject H1o in favor of H1a, i.e., using TeamWATCH does help developers detect and resolve potential conflicts earlier.

In the experimental group, we observed that two teams did not pay attention to the smoke emitted from the files in the TeamWATCH visualization when they were working on the first task; thus, the team member who checked in the changes late encountered a conflict during the check-in time. Then, the teams realized the importance of the hints provided by TeamWATCH and leveraged it to coordinate together to avoid merge conflicts in the subsequent four tasks. Another team did not realize it until the second task. Overall, the five teams in the experimental group could use IM to coordinate their work to avoid the merge conflicts.

The main strategy adopted by most of the teams in the experimental group is as follows: first, one team member checks-in the local changes for a file; then, the other member syncs to the repository to get the changes for that file and then starts working on the task related to that file. Sometimes, they even switched the order of the tasks that they were working on to avoid conflicts. In addition, an excerpt of the chat logs from one team in the experimental group is given below.

**Developer 1**: *just modified chapter 1*, *sync now*

**Developer 2**: *changed chapter 1 I think it worked check commit*!!!!!!!!!!!!!!!!!!!!!!

In the control group, we observed that most teams only detected conflicts during the check-in time and had to resolve the merge conflicts. The only exception was one team who coordinated at the beginning of the experiment regarding how to avoid conflicts by exchanging the tasks assigned to them with each other and by suggesting that one team member start from the last task and work backwards while the other member worked forward. The abovementioned team figured out at the beginning that there would be potential conflicts, which, in a sense, proved that the TeamWATCH tool would be very helpful to them since it could help them detect and avoid potential conflicts as soon as they occurred. Two teams were able to figure out how to avoid the conflicts in the last one or two tasks and coordinate via IM to achieve it.

#### 5.2.3 Analysis of maintaining group awareness in general

The ability to detect and resolve conflicts earlier is one way to verify that the TeamWATCH tool can help users to better maintain group awareness. In this section, we look for other proof mainly from the post-experiment survey data. The survey data were collected via the online survey website surveymonkey.com, and some participants did not fill out (or submit) their surveys before leaving the experiment. In total, nine subjects from the treatment group and eleven subjects from the control group submitted their post-experiment surveys.

For the survey question “Were you aware of the status of your teammate (e.g., what was he/she working on at any particular moment)?”, eight out of nine (i.e., 88.9%) responders from the experimental group thought that they were aware of their team member’s status, while only six out of the eleven (54.5%) responders from the control group were aware of their team member’s status.

For the survey question “Did TeamWATCH help you to better maintain group awareness (i.e., to know the status of the project artifacts and the status of your team members)? If yes, please give an example.”, all seven responders from the experimental group gave positive answers, and most of them also gave an example, some of which are shown below

- *yes!* *helped me to find out what my team member was working on*
- It helped when someone was actually committing
- yes checking if a teammate had edited a file I was editing
- *yes*, *I could see when I was making revisions as well as my partner*

The post-experiment survey designed specifically for the experimental group (since the questions are all related to TeamWATCH) also asked the subjects how often they used TeamWATCH during the experiment; most of them said they used it quite often.

- *I used it as often as possible to see who changed the file and when they did so*.
- *pretty often*, *checked the visualization of what was happening*
- the entire time

When asked which features of TeamWATCH were the most useful to them, the answers from the subjects fell into the following two categories

- The visualization of who is editing/modifying which file
- *The filter or search functionality*.

### 5.3 Internal validity

#### 5.3.1 Subjects

The number of subjects in the experiment was low. The results of the comparison between the two groups would be more accurate if we could recruit more subjects.

Subjects were CS graduate and undergraduate students, and they had little experience with version control systems such as CVS and integrated development environments such as Eclipse compared to experienced professional software engineers. However, subjects were given CVS and Eclipse assignments before the experiment and instructions in the experiment sheet to help them get familiar with the tools.

Subjects were randomly assigned to either the experimental group or the control group. The pre-survey results showed that both groups shared the same (i.e., approximately 6 months) amount of experience with version control systems.

#### 5.3.2 Contextual project

A text editing project, instead of a software project, was chosen to mitigate the risk of the experience of the subjects influencing the experimental results, as explained in the previous section. Since the subjects were all computer science students, a book on a computer science topic (i.e., the Internet) was chosen to achieve the same level of topic familiarity for all subjects.

#### 5.3.3 Tasks

To mitigate the threat that the experimental task design may be biased to the advantage of TeamWATCH, the most common conflicts that developers can encounter in daily work (i.e. direct conflicts such that one developers add the new functionalities while the other refactor the existing functionalities in the same artifact [16]) were selected, and all the tasks could be completed using only the Eclipse CVS plug-in (i.e., with or without TeamWATCH). Furthermore, we did not record nor compare the time required to answer these questions between the two groups.

### 5.4 External validity

#### 5.4.1 Subjects

The subjects in both groups were computer science students. Their experience in software development was different from that of real-world professional software developers, who are the ultimate target audience of this prototype tool.

#### 5.4.2 Contextual project

The experimental project chosen in this study was a small-scale text editing project with 19 files and approximately 200 revisions developed by four editors (including the existing two editors who made all the revisions to establish the basis for this experiment and the two subjects who finished the editing tasks). This does not really simulate a real software development project; however, as explained in the previous section, it was introduced to let the subjects focus on the evaluation of the tool instead of spending most of their time determining how to finish the coding tasks. Although large software systems were not simulated in the experiment, even for the large software system, the developers will be mostly interested in the component they are working on or depending on, and its visualization can be customized via TeamWATCH. While further evaluation of TeamWATCH’s effectiveness for larger-sized software projects developed by a larger team is desirable, the current results of this experiment are informative and worth sharing with the software engineering community.

#### 5.4.3 Tasks

The five tasks (including the coding subtasks and the CVS historical information questions) used in the experiments did not cover all types of tasks/questions developers may encounter in collaborative work. Nevertheless, these code tasks were designed to cover different types of coding changes that might introduce potential conflict, and the questions were designed based on the “who, what, when, where, how” criteria and represented the most frequently asked software source code historical questions [23].

#### 5.4.4 TeamWATCH

The experiment designers were aware that TeamWATCH did not represent all the awareness tools. Therefore, even though the experiment could be a fair evaluation of the TeamWATCH tool, generalization of this specific outcome regarding TeamWATCH for software awareness in general could be a threat to the validity of the general result. However, better tool designs will likely only produce even better results than what has been shown with this version of TeamWATCH.

## 6. Conclusions and future work

Although coworker and artifact awareness information is essential for collaboration among software developers in a team, there is inadequate tool support to help them acquire it. TeamWATCH, a workspace awareness tool based on a 3-D city metaphor, was built to support visualizing both historical and real-time awareness info in a shared common view. In a controlled user experiment, we specifically evaluated TeamWATCH to test its effectiveness in enabling users to detect potential conflicts and to collaborate to resolve conflicts earlier to avoid merge conflicts during check-in. The statistically significant results showed that TeamWATCH helped users detect and resolve a larger number of conflicts earlier when compared to users without any workspace awareness support. This clearly demonstrated the effectiveness of TeamWATCH in reducing the effects of conflicts and thus improving developers’ efficiency in software development. It also provided qualitative evidence of the effectiveness of TeamWATCH in maintaining group awareness.

In the future, we plan to add the support of indirect conflict detection, integrate with more version control repositories such as Git, improve the UI interaction of TeamWATCH by leveraging Nature User Interface tools such as Kinect and by developing a mobile version of TeamWATCH with the emerging of mobile awareness visualization tools [49,50], and evaluate it with a real software development team.

## Supporting information

S1 Table Experiment tasks. (DOCX)

S1 File Conflict detection results. (XLSX)

S2 File PreSurvey—Control group responses. (XLSX)

S3 File PreSurvey–experimental group responses. (XLSX)

S4 File PostSurvey–control group responses. (XLSX)

S5 File PostSurvey–experimental group responses. (XLSX)
