# Supplementary material for: TeamWATCH: Visualizing development activities using a 3-D city metaphor to improve conflict detection and team awareness
Source: PLoS One. 2018 Mar 20;13(3):e0193562. doi: 10.1371/journal.pone.0193562 (PMC5860726; doi:10.1371/journal.pone.0193562)
Supplement: S1 Table — (DOCX) [file pone.0193562.s001.docx]

# **S1 Table: Experimental tasks**

| **Tasks for Subject A in each team of two** | **Tasks for Subject B in each team of two** |
| --- | --- |
| 1. CVS question: Who last revised Chapter 1? __________ 2. In Chapter 1, switch the order of the last two paragraphs in section 1.1, and move the following text: “There are the basics. Now on to the Net!” to a new paragraph. | 1. CVS question: Who last revised Chapter 1? _______________ 2. In Chapter 1, search for the first occurrence of the texts “8-1-N” and “7-1-E”, add the text “(which stands for "8 bits, 1 stop bit, no parity" -- yikes!)” immediately after “8-1-N”, and add the text “(7 bits, 1 stop bit, even parity)” immediately after “7-1-E”. |
| 1. In Chapter 7, search for and correct the following number errors 2. Modify the text “50,000-byte file” to “500,000-byte file” 3. Modify the text “24000-baud modem” to “2400-baud modem” 4. Modify the text “20,000 books” to “10,000 books" 5. Switch the years in the following text:   In 1994, the project uploaded an average of four books a month to its ftp sites; in 1993, they hope to double the pace.   1. CVS question: When did the editor “en” last revise Chapter 7? __________ 2. In Chapter 7, insert the following text at the end of section 7.10 FYI   The comp.sys.ibm.pc.digest and comp.sys.mac.digest newsgroups provide  information about new MS-DOS and Macintosh programs as well as answers to questions from users of those computers. | 1. In Chapter 7, insert the following text at the end of section 7.10 FYI   In the comp.virus newsgroup on Usenet, look for postings that list  ftp sites carrying anti-viral software for Amiga, MS-DOS, Macintosh,  Atari and other computers.   1. CVS question: When did the editor “en” last revise Chapter 7? _______________ 2. In Chapter 7, reverse the first name and last name in the following text: “Emtage Alan, Heelan Bill and Deutsch Peter” |
| 1. CVS question: When was the last revision of Chapter 8 committed? __________ 2. At the end of section 8.1, the order of the following two commands “ms-dos or macintosh” and “ms-dos and macintosh” is wrong. Correct it by switching them. | 1. CVS question: How many editors contributed to Chapter 5? _______________ 2. In Chapter 5, insert the following two Command-BitnetList-UsenetList triples where two <<ADD COMMAND>>, <<ADD BITNETLIST>>, <<ADD USENETLIST>> strings are found. 3. sub NEW-LIST Your Name, [listserv@ndsuvm1.bitnet](mailto:listserv@ndsuvm1.bitnet), bit.listserv.new-list   sub INFONETS Your Name, [info-nets-request@think.com](mailto:info-nets-request@think.com), bit.listserv.info-nets |
| 1. CVS question: How many revisions has Chapter 2 received so far? __________ 2. In Chapter 2, section 2.1, search for and replace the text “<<ADD DOMAIN SUFFIX EXAMPLES>>” with the below domain suffix examples and then order them alphabetically   .com for businesses  .org for non-profit organizations  .gov and .mil for government and military agencies  .net for companies or organizations that run large networks. | 1. In Chapter 2, section 2.1, insert the following paragraph immediately after <<ADD DOMAIN SUFFIX EXAMPLES>>   Sites in the rest of the world tend to use a two-letter code that  represents their country. Most make sense, such as .ca for Canadian  sites, but there are a couple of seemingly odd ones. Swiss sites end  in .ch, while South African ones end in .za. Some U.S. sites have  followed this international convention (such as well.sf.ca.us).   1. CVS question: How many revisions has Chapter 2 received so far? ______________ 2. In Chapter 2, switch the universities (i.e., the content in the brackets immediately after the email addresses) that [tomg@unm.edu](mailto:tomg@unm.edu) and [tomg@umn.edu](mailto:tomg@umn.edu) represent |
| 1. CVS question: Who has contributed the most revisions to Chapter 10? _______ 2. In Chapter 10, remove section 10.7 The all-knowing Oracle, rename section 10.8 FYI to 10.7, and then update the Table of Contents accordingly | 1. CVS question: Who has contributed the most revisions to Chapter 10? _______ 2. Insert the following text immediately after section 10.7 as the new section 10.8, rename the original section 10.8 as section 10.9, and then update the Table Of Contents accordingly   10.8 WHEN THINGS GO WRONG  * You get back an error message that your fax could not be delivered.  With TPC, that could mean one of two things. Either you tried sending a fax to an area not covered by TPC or you made a mistake converting the fax number into a TPC address. Double-check both the list of TPC coverage areas and the address you created. |
